# Honeycomb Constructs in the La–Ni Intermetallics: Controlling Dimensionality via *p*-Element Substitution

**DOI:** 10.1021/acs.inorgchem.3c00502

**Published:** 2023-09-07

**Authors:** Vitalii Shtender, Volodymyr Smetana, Jean-Claude Crivello, Łukasz Gondek, Janusz Przewoźnik, Anja-Verena Mudring, Martin Sahlberg

**Affiliations:** † Department of Chemistry−Ångström Laboratory, Uppsala University, Box 538, 751 21 Uppsala, Sweden; ‡ Department of Materials and Environmental Chemistry, Stockholm University, Svante Arrhenius väg 16c, 10691 Stockholm, Sweden; § Univ Paris Est Creteil, CNRS, ICMPE, UMR7182, 2 rue Henri Dunant, 94320 Thiais, France; ⊥ Faculty of Physics and Applied Computer Science, AGH University of Science and Technology, Mickiewicza 30, 30-059 Krakow, Poland

## Abstract

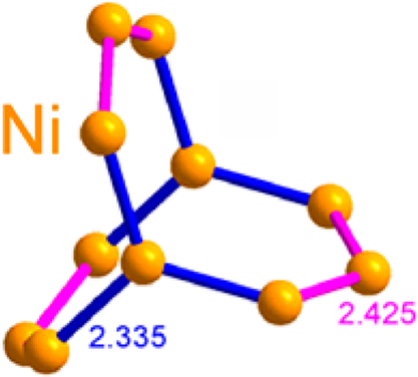

The new ternary compounds La_15_Ni_13_Bi_5_ and La_9_Ni_8_Sn_5_ were obtained by arc melting under argon from appropriate amounts of the elements and subsequent annealing at 800 °C for 2 weeks. Single-crystal X-ray diffraction reveals that they represent two new structure types: La_15_Ni_13_Bi_5_ crystallizes in the hexagonal space group *P*62*m* [*hP*33, *a* = 14.995(3), *c* = 4.3421(10) Å, *V* = 845.5(4) Å^3^, *Z* = 1] and La_9_Ni_8_Sn_5_ in *P*6_3_/*m* [*hP*88, *a* = 23.870(15), *c* = 4.433(3) Å, *V* = 2187(3) Å^3^, *Z* = 4]. The crystal structures of both compounds are characterized by hexagonal honeycomb-based motifs formed by Ni and Sn that extend along the *c* axis. The building motif with its three-blade wind turbine shape is reminiscent of the organic molecule triptycene and is unprecedented in extended solids. First-principles calculations have been performed in order to analyze the electronic structure and provide insight into chemical bonding. They reveal significant electron transfer from La to Ni and the respective *p*-element, which supports the formation of the polyanionic Ni-*p*-element network. DFT calculations suggest paramagnetic-like behavior for both compounds, which was confirmed by magnetic measurements.

## Abstract 2

The new ternary intermetallics La_15_Ni_13_Bi_5_ and La_9_Ni_8_Sn_5_ have been studied, leading to the discovery of honeycomb-based motifs in both compounds. DFT and phonon calculations confirmed the lattice stability of the honeycomb constructs.

## 1 Introduction

Bismuth and tin-based intermetallic compounds are interesting for many technologies and high-tech applications. They own many unique and desirable properties for optoelectronics, solders, fusible alloys, substitutes of lead, thermoelectrics, superconductors, and electronic device applications.^1−3^ Superconductivity has been observed in many *p*-elements, such as Sn, and in their binary intermetallic compounds, like in the La–Sn system.^4−6^ Nb_3_Sn shows excellent superconducting performance at lower temperatures and under high magnetic fields.^7^ Pd_3_Bi has been applied in catalysis;^8^ bismuth tellurides found applications as superconductors, as advanced thermoelectric materials, and in mobile refrigerators or infrared detectors.^9^ La–Ni alloys feature enhanced hydrogen sorption, and through the addition of certain *p* elements, their mechanical resistance is improved, as in LaNi_5_Sn, although capacity is sacrificed.^10,11^

Interestingly, the ternary La–Ni–Sn system has been only partially investigated, resulting in a preliminary phase diagram at 400 °C.^12^ So far, eight ternary compounds have been confirmed: LaNi_5_Sn (CeNi_5_Sn type),^13^ LaNi_4_Sn_2_ (KAu_4_Sn_2_ type),^14^ LaNi_2_Sn_2_ (CaBe_2_Ge_2_ type),^15^ La_3_Ni_2_Sn_6_ = LaNi_0.7_Sn_2_ (CeNiSi_2_ type),^16^ La_3_Ni_2_Sn_7_ (La_3_Co_2_Sn_7_ type),^17^ La_3_Ni_8_Sn_16_ = La_4.87_Ni_12_Sn_24_ (Gd_4_(Gd_0.5_Sn_0.5_)Ni_12_Sn_24_ type),^18^ LaNiSn (TiNiSi type),^19^ and La_5_Ni_24_Sn = LaNi_4.8_Sn_0.2_ (essentially a solid solution based on LaNi_5_) (CaCu_5_ type).^20^ LaNiSn_3_ has been mentioned, but no crystal structure has ever been reported.^21^ LaNiSn shows polymorphism under pressure switching from the TiNiSi type to the ZrNiAl type.^22^ All ternary compounds in this system (up to date) are La-poor, reaching a maximum of 33.3 at. % of La.^21^ The related La–Ni–Bi system has been even more poorly investigated, with only three ternary compounds reported so far: La_2_NiBi (La_2_NiSb-type),^23^ LaNi_2–*x*_Bi_2_ (CaBe_2_Ge_2_-type for *x* = 0.5 and CuHfSi_2_-type for *x* = 1.16),^24,25^ and La_3_Ni_3_Bi_4_ (Y_3_Au_3_Sb_4_-type),^26^ and no phase equilibrium has been established.

This prompted us to close the knowledge gap and reinvestigate the ternary La–Ni–X systems with X = Sn, Bi. During these explorations, two new compounds were discovered with interesting structural features, namely, hexagonal honeycomb motifs extending along the 3(6)-fold axis, La_15_Ni_13_Bi_5_ and La_9_Ni_8_Sn_5_. These motifs are reminiscent of the three-blade wind turbine shape of triptycene and are unprecedented in extended solids. In the present work, we report on their crystal and electronic structures, analyze the chemical bonding, and discuss relationships with other intermetallics.

## 2 Methodology

### 2.1 Experimental Methods

La/M/Ni (M = Bi and Sn) alloys (1 g each) with the molar ratios of the elements 15:13:5 and 9:8:5, respectively, were synthesized by arc-melting of high purity La (99.9%), Ni (99.99%), Bi (99.9%), and Sn (99.99%) from Alfa Aesar. An excess of Bi (1 wt %) was used to compensate for losses during the melting. Oxygen contamination was minimized by flushing the furnace five times with Ar and by melting a Ti getter before melting the sample. The sample ingots were turned over and remelted three times to promote homogeneity. The ingots then were wrapped in Ta-foil, placed in a quartz tube, and sealed under a vacuum for later annealing at 800 °C for 2 weeks. Subsequently, samples were quenched in cold water.

An alternative method has also been used to obtain crystals of better quality. According to the binary La–Ni phase diagram, alloys in the region of 50–80 at. % of La exhibit relatively low melting points below 700 °C. Therefore, La_15_Ni_13_ and La_9_Ni_8_ ligatures were prepared by arc melting, crushed, and mixed with appropriate amounts Bi and Sn. The mixtures were loaded into Ta tubes, sealed under an Ar atmosphere, and subsequently placed in stainless steel tubes to protect from oxidation. The samples were slowly heated (10 h) up to 1000 °C, kept for 1 h, and cooled at 3 °C/h down to 500 °C. For cooling to room temperature, the furnace was switched off.

Intensity data sets for powder X-ray diffraction (PXRD) were recorded at room temperature using a Bruker D8 X-ray diffractometer with a Lynx-eye position sensitive detector and Cu Kα radiation on a zero-background single-crystal Si sample holder. Phase analyses (Figure S1 in the Supporting Information) using the Rietveld method of the powder X-ray data were performed using TOPAS v6 software.^27^

Single-crystal X-ray diffraction (SXRD) data were collected at 293 K on a Bruker D8 Venture diffractometer (Bruker, USA; Photon 100 CMOS detector, IμS microfocus source: Mo Kα radiation, λ = 0.71073 Å, 2–5). Intensity data sets of reflections and scaling were integrated within the APEX3 software package by using SAINT.^28^ Absorption corrections were conducted with SADABS^29^ and crystal structure solutions with SHELXT.^30^ For subsequent difference Fourier analyses and least-squares refinements, SHELXL-2013^31^ was used. The mixed Sn1/Ni1 position in La_9_Ni_8_Sn_5_ has initially been refined with independent occupation, resulting in a 1:2 ratio within 1 sigma. Therefore, the occupations of the Sn and Ni components have been restricted to 1/3 and 2/3. Such occupation matches well with the neighboring positionally disordered La2–La3–La4 positions explaining the somewhat higher ADPs. The experimental details of the crystal structure determination and refinement as well as the atomic coordinates are collected in Tables 1 and 2. Studied crystals were well stable in air (checked after 1 month), but the alloys in general could be slightly affected by hydrolysis (a minor amount of La(OH)_3_ was identified from the PXRD).

**Table 1:** Crystallographic Data and Experimental Details of the Structure Determination for La_15_Ni_13_Bi_5_ and La_9_Ni_8_Sn_5_^a^

Empirical formula	La_15_Ni_13_Bi_5_	La_9_Ni_8_Sn_5_
CSD	2241021	2241022
Composition in at.% from SCXRD	La_45.46_Ni_39.39_Bi_15.15_	La_40.91_Ni_36.36_Sn_22.73_
Composition at.% from EDX	La_45.9(4)_Ni_38.7(4)_Bi_15.4(1)_	La_42.3(9)_Ni_35.9(8)_Sn_21.8(4)_
Structure type	own	own
Formula weight, *M*_r_ (g/mol)	3891.78	2313.32
Space group (No.)	*P*62*m* (189)	*P*6_3_/*m* (176)
Pearson symbol, *Z*	*hP*33, 1	*hP*88, 4
*a*, Å	14.995(3)	23.87(1)
*c*, Å	4.342(1)	4.433(3)
*V*, Å^3^	845.5(4)	2187(3)
Flack parameter	0.058(19)	–
Calculated density, ρ (g·cm^–3^)	7.64	7.03
Absorption coefficient, μ (mm^–1^)	51.38	29.39
Theta range for data collection (deg)	1.568–27.494	0.985–25.469
*F*(000)	1634	3948
Range in *h k l*	–19 ≤ *h* ≤ 19,	–28≤ *h* ≤ 28,
	–19 ≤ *k* ≤ 19,	–26 ≤ *k* ≤ 26,
	–5 ≤ *l* ≤ 5	–5 ≤ *l* ≤ 5
Total no. of reflections	10252	10649
No. of independent reflections	773 (*R*_eq_ = 0.0411)	1418 (*R*_eq_ = 0.0715)
Parameters	42	100
Goodness-of-fit on *F*^2^	1.262	1.273
Final *R* indices [*I* > 2σ(*I*)]	*R*_1_ = 0.0223;	*R*_1_ = 0.0616;
	w*R*_2_ = 0.0583	w*R*_2_ = 0.1244
*R* indices (all data)	*R*_1_ = 0.0223;	*R*_1_ = 0.0768;
	w*R*_2_ = 0.0583	w*R*_2_ = 0.1293
Extinction coefficient	0.00062(12)	0.00007(2)
Δρ_max/min_ (e·Å^–3^)	2.231/–1.874	3.696/–2.796

a Experiments were carried out at 296 K with Mo Kα radiation.

**Table 2:** Atomic Coordinates and Equivalent Isotropic Displacement Parameters for La_15_Ni_13_Bi_5_ and La_9_Ni_8_Sn_5_

Atom	Site	*x*	*y*	*z*	SOF	*U*_eq_*100 (Å^2^)
**La**_**15**_**Ni**_**13**_**Bi**_**5**_						
La1	6*k*	0.8573(1)	0.39297(9)	1/2	1	1.01(3)
La2	6*j*	0.8611(1)	0.6185(1)	0	1	1.10(3)
La3	3*g*	0.1559(1)	0	1/2	1	1.02(4)
Ni1	3*g*	0.7725(3)	0	1/2	1	1.6(1)
Ni2	3*g*	0.6120(3)	0	1/2	1	1.9(1)
Ni3	3*f*	0.8447(3)	0	0	1	1.38(9)
Ni4	3*f*	0.5291(3)	0	0	1	1.32(9)
Ni5	1*a*	0	0	0	1	1.4(2)
Bi1	3*f*	0.34021(8)	0	0	1	0.96(3)
Bi2	2*c*	2/3	1/3	0	1	0.98(3)
**La**_**9**_**Ni**_**8**_**Sn**_**5**_						
La1	6*h*	0.0312(1)	0.4174(1)	1/4	1	1.80(5)
La2	6*h*	0.1088(1)	0.5954(1)	1/4	1	1.81(5)
La3	6*h*	0.1930(1)	0.4342(1)	1/4	1	1.80(5)
La4	6*h*	0.2647(1)	0.2970(1)	1/4	1	1.97(5)
La5	6*h*	0.5704(1)	0.2551(1)	1/4	1	1.89(5)
La6	6*h*	0.2728(1)	0.1485(1)	1/4	1	3.05(6)
Sn1/Ni1	6*h*	0.0107(2)	0.1907(2)	1/4	0.333/0.667	4.3(1)
Sn2	6*h*	0.4136(1)	0.1265(1)	1/4	1	1.97(6)
Sn3	6*h*	0.5164(1)	0.3645(1)	1/4	1	1.52(6)
Sn4	6*h*	0.0635(3)	0.1171(3)	1/4	0.333	0.61(9)
Sn5	6*h*	0.1040(3)	0.1117(3)	1/4	0.333	0.61(9)
Sn6	6*h*	0.1192(3)	0.1620(3)	1/4	0.333	0.61(9)
Ni2	6*h*	0.0213(7)	0.0333(6)	1/4	0.333	1.9(3)
Ni3	6*h*	0.0693(2)	0.3156(2)	1/4	1	2.3(1)
Ni4	6*h*	0.2236(2)	0.5758(2)	1/4	1	2.4(1)
Ni5	6*h*	0.3818(2)	0.2882(2)	1/4	1	2.0(1)
Ni6	6*h*	0.5290(2)	0.0302(2)	1/4	1	2.2(1)
Ni7	2*c*	1/3	2/3	1/4	1	2.8(2)

The microstructure was evaluated with a Zeiss Merlin SEM instrument equipped with a secondary electron (SE) detector and an energy-dispersive X-ray (EDX) spectrometer. The samples for electron microscopy analysis were prepared by standard metallographic techniques by grinding with SiC paper. For the final polishing, a mixture of SiO_2_ and H_2_O was used. SEM images together with EDX compositions are presented in Figure S2.

Magnetic measurements were conducted by using a Physical Property Measurement System (PPMS, Quantum Design, USA). Vibrating Sample Magnetometer (VSM) options were utilized to measure zero-field-cooled (ZFC) and field-cooled (FC) magnetization between 2 and 300 K in static fields (DC) up to 7 T. Isothermal magnetization was acquired in applied fields up to 7 T. Polycrystalline samples (50–65 mg) were filled into polypropylene (PP) capsules, which were mounted into a brass sample holder.

### 2.2 First-Principles Calculations

The electronic structure of La_15_Ni_13_Bi_5_ and La_9_Ni_8_Sn_5_ were calculated in the DFT framework in a pseudopotential approach in the projector augmented-wave (PAW) method with collinear spin polarization using the VASP package,^32,33^ but without spin–orbital coupling. The generalized gradient approximation (GGA) described with the PBE function^34^ was used with a cutoff energy of 600 eV, within a high k-mesh density (about 200 points in the irreducible Brillouin zone). Preserving the original crystal symmetry, each structure was fully relaxed by several relaxation schemes (volume, cell shape as the *c*/*a* ratio, and atomic positions).

Based on the experimental observation, the crystal structures of La_15_Ni_13_Bi_5_ and La_9_Ni_8_Sn_5_ were described using hexagonal cells (see section 3.1 for crystallographic details). Whereas La_15_Ni_13_Bi_5_ was considered in the ordered structure (33 atoms/cell), La_9_Ni_8_Sn_5_ was studied within a disordered description. To deal with the (2/3, 1/3) occupation by (Ni, Sn) on the 6*g* (*x* = 0.01090, *x*, *z* = 1/4) site for La_9_Ni_8_Sn_5_, several “locally ordered” supercells representing a wide range of possibilities have been considered (2 × 2 × 1 cell leading to 88 atoms/cell). After relaxation, the most stable arrangement corresponds to the one that maximizes the number of Ni pairs. Therefore, this was used for further discussion.

A careful relaxation was performed so that the convergence of Hellmann–Feynman forces was better than 0.05 meV Å^–1^. Then, the phonon calculations are carried out in the harmonic approximation for La_9_Ni_8_Sn_5_ from the supercell approach (1 × 1 × 2) with the finite displacement method^35^ using the Phonopy code.^36^ The electron localization function (ELF),^37−39^ which defines a region of space that can be associated with an electron pair, was computed for the valence electrons and is represented using the VESTA package.^40^ Charge transfers are computed using Bader’s prescription.^41^

## 3 Results and Discussion

### 3.1 Crystal Structure Peculiarities

La_15_Ni_13_Bi_5_ crystallizes in the hexagonal space group *P*62*m* [*hP*33, *a* = 14.995(3), *c* = 4.342(1) Å, *V* = 845.5(4) Å^3^, *Z* = 1], while La_9_Ni_8_Sn_5_ crystallizes in *P*6_3_/*m* [*hP*88, *a* = 23.870(15), *c* = 4.433(3) Å, *V* = 2187(3) Å^3^, *Z* = 4]. Both compounds represent their own structure types, though exhibiting plenty of similar structural motifs. For La_15_Ni_13_Bi_5_, a fully ordered structure is observed, while La_9_Ni_8_Sn_5_ exhibits complex positional and occupational disorder.

**Figure 1:**
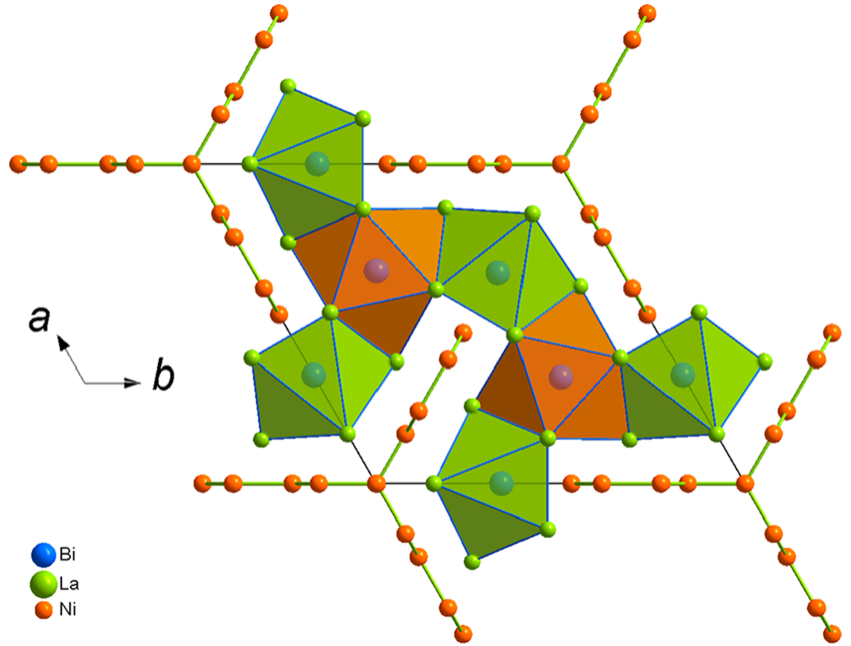
Projection of the crystal structure of La_15_Ni_13_Bi_5_ along [001], with outlined Ni sticks and Bi polyhedra.

**Figure 2:**
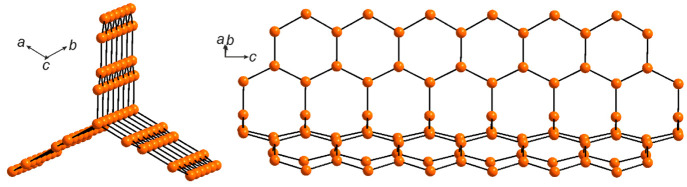
Ni sticks in the crystal structure of La_15_Ni_13_Bi_5_ extending along the *c* axis.

The crystal structure of La_15_Ni_13_Bi_5_ is characterized by pseudo-1D motifs of Ni encapsulated in the La/Bi tunnels. The latter are composed of two types of fused Bi@La_*x*_ polyhedra, i.e., alternating tricapped Bi@La_9_ and bicapped Bi@La_8_ trigonal prisms (Figures 1 and S5). The tricapped prisms are regular, while the bicapped ones also include a (smaller) Ni atom in the Bi coordination sphere at a reasonable interatomic distance, i.e. (Bi@La_8_Ni), leading to significant distortion of the central trigonal prism. Although there may be non-negligible interactions, we consider the Ni formations in the structure independently for structural clarity. The Ni atoms form a stick that extends along the *c* axis (Figure 2). The single building unit of the stick bears a structural analogy to organic aromatic molecules like triptycene that are reminiscent of a three-blade wind turbine. To the best of our knowledge, such a structural motif has never been reported in extended solids. Each turbine blade consists of one full row of fused Ni_6_ hexagons, while the second (central) one is missing its vertices (Figure S3). Instead, two lower Ni atoms are shared with two identical blades. The Ni–Ni distances within the hexagons are 2.414–2.498(9) Å, while those connecting the blades are a little shorter, 2.334(8) Å, all below or around the sum of the covalent radii.^42^ The outer Ni vertices connect with Bi atoms from irregular prisms (*d*_Ni–Bi_ = 2.836(8) Å). All other Ni positions have a coordination number of 9 and a coordination sphere of practically ideal tricapped trigonal prism Ni@Ni_3_La_6_ (Figure S4). La_6_ prisms lie normal to the blade plane, fully wrapping the Ni blades. The axial Ni atoms have identical coordination, although the La_6_ prism around it is oriented along the *c* axis. La–Ni distances in the structure are in the range of 2.880–3.189(6) Å, suggesting some charge transfer.

**Figure 3:**
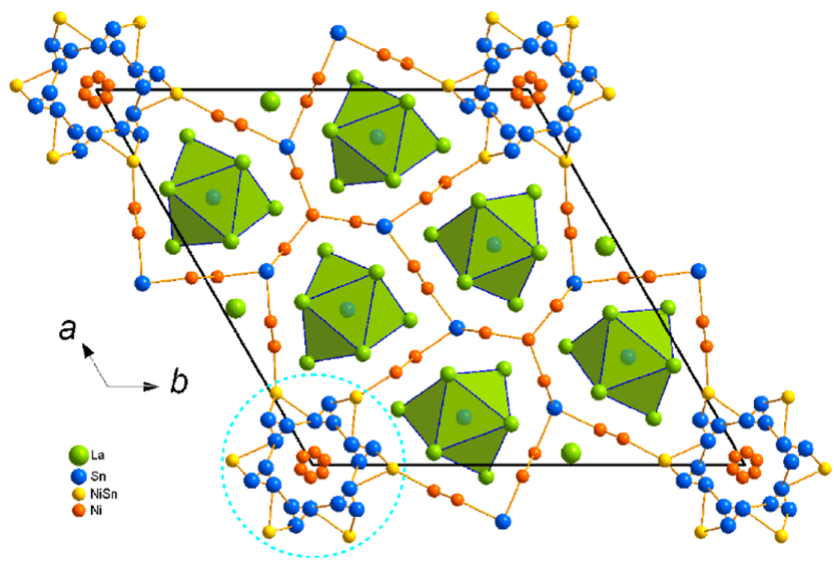
Projection of the crystal structure of La_9_Ni_8_Sn_5_ along [001], with outlined Ni–Sn nets and Sn polyhedra. Sites within dashed blue circles are compositionally (Sn and Ni) or occupationally (Ln and Ni) disordered.

**Figure 4:**
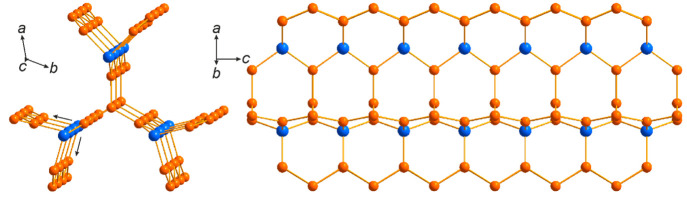
Ni–Sn framework in the crystal structure of La_9_Ni_8_Sn_5_ extending along the *c* axis.

La_9_Ni_8_Sn_5_ was detected during an attempt to explore possible isostructural representatives of La_15_Ni_13_Bi_5_. No such compounds could be observed, though the discovered *p*-element-richer La_9_Ni_8_Sn_5_ exhibits structural similarities, but with enhanced complexity partially due to extensive disorder. Contrary to La_15_Ni_13_Bi_5_, La_9_Ni_8_Sn_5_ is characterized by a polyanionic Ni/Sn network, forming tunnels that encapsulate Sn-centered La polyhedra, Sn@La_9_ (Figures 3 and S6). They are constituted by a Sn-centered La_6_ trigonal prism capped by three La atoms and are reminiscent of the Bi@La_9_ polyhedra observed in La_15_Ni_13_Bi_5_. The motif formed by Ni and Sn now extends in three directions though keeping the same basis—a three-blade turbine of Ni and Sn atoms (Figure 4). Further expansion beyond the Sn goes in two directions, forming a nearly 90° angle between the branches (Figure 4, black arrows). Each of the branches is represented by Ni zigzag chains leading to a row of Ni_4_Sn_2_ fused distorted hexagons. One of the chains is shared and responsible for connection with an identical basis leading to the formation of the framework. The other one goes toward the highly disordered area around the *c* axis. Interestingly, Ni–Ni distances in the basis are the shortest—2.429(9) Å—while those in the zigzag chains are 2.616(4) Å due to geometric factors and higher influence of the competing interactions with the Sn atoms. Ni–Sn contacts in the ordered framework are in the range of 2.723–2.789(5) Å.

The area around the *c* axis is highly disordered (Figure 3, dashed circle), though disorder can be rationalized. Such disorders occur mainly due to geometric reasons and are typical for hexagonal lanthanide compounds with transition and post-transition elements and a short *c* axis.^43−45^ Three sites are involved: the central Ni position is split over three locations with the total occupation of a hypothetical 2*a* position right on the *c* axis. The second layer includes heavily positionally disordered Sn 6*h* positions also split over three locations, while the last one is represented by mixed Ni/Sn occupation (0.67 Ni/0.33 Sn). Such composition assignment is confirmed by the results of EDX analysis, as well as interatomic distances to the neighboring Ni positions being in the upper range for the other observed Ni–Ni contacts in the structure and nearly equal to the sum of the corresponding covalent radii (2.58(1) Å).

As we mentioned before, La_15_Ni_13_Bi_5_ has no direct analogues in the literature. Considering only the central part of the triangular motifs, geometrically similar fused hexagonal motifs could only be found in La_10_Co_7_Ga_3_,^46^ though including also La atoms and offering no further expansion of the honeycomb network. Compositionally similar Ce_15_Ni_4_Si_13_^47^ exhibits a completely different packing, though, based on fused trigonal prismatic building blocks of the lanthanide atoms based on the ZrNiAl-type.^22^ Indeed, Ln_(*n*+1)(*n*+2)_Ni_*n*(*n*–1)+2_Si_*n*(*n*+1)_^43^ exhibit features observed in both La_15_Ni_13_Bi_5_ and La_9_Ni_8_Sn_5_. Extensive positional disorder observed in La_9_Ni_8_Sn_5_ is rather typical for related hexagonal structures, particularly those with a short *c* axis parameter.^47−50^ While polyanionic tunnels with encapsulated cations are popular in intermetallics,^51^ encapsulated mixed motifs are less common. For instance, complex mixed formations inside Au/In tunnels have been observed in Ca_3_Au_3_In.^52^ Capped trigonal prismatic motifs both isolated and fused are quite common in binary intermetallics.^53−55^

### 3.2 Electronic Structure

Previous investigations of the electronic structure of La–Ni-based compounds with additional *p*-elements, such as Sn, have shown that the occupied states of the valence band are mainly composed of flat 3*d*-Ni bands hybridized with 6*s*-La and 5*p*-Sn states, in which the 5*s*-Sn states are found at lower energy separated by a gap.^56^

**Figure 5:**
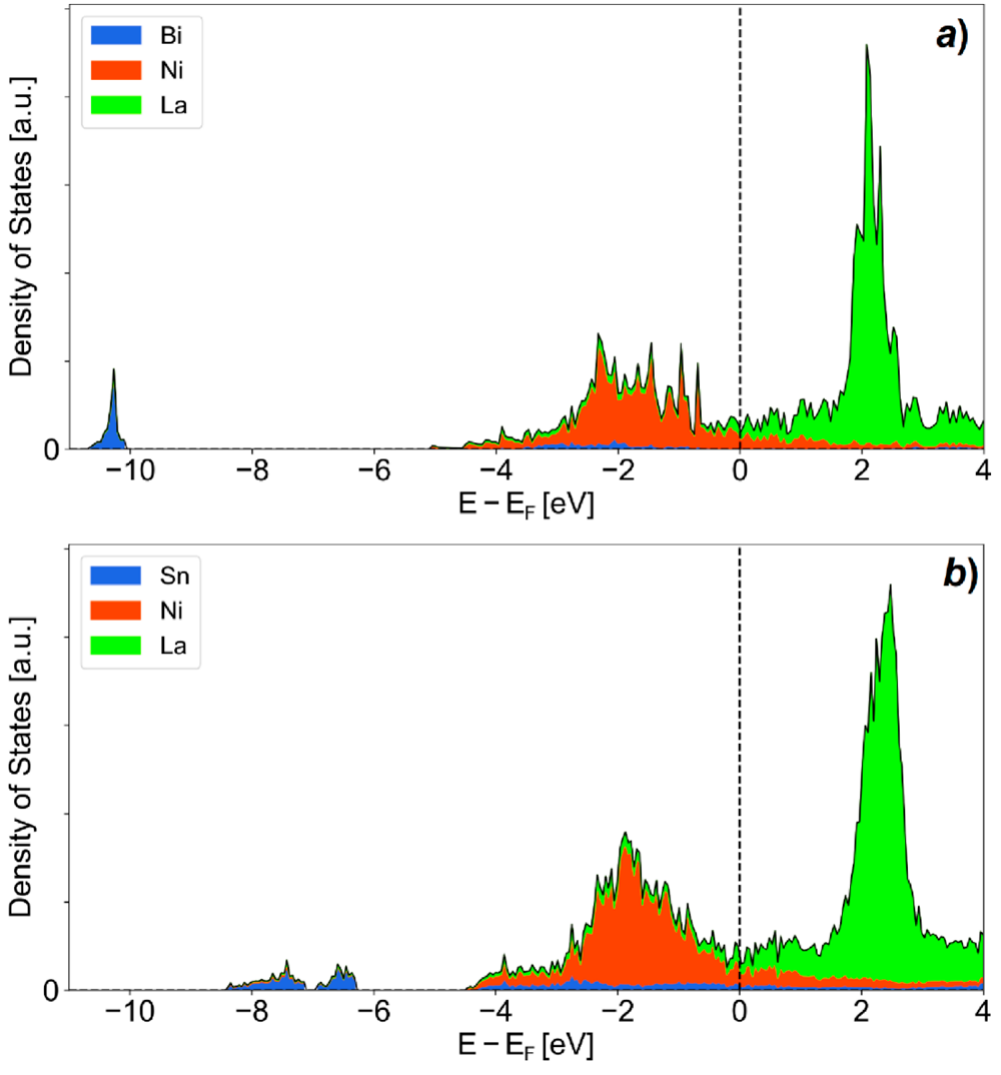
Total density of state (black line) with site-projected contributions of all atoms in La_15_Ni_13_Bi_5_ (a) and La_9_Ni_8_Sn_5_ (b). The Fermi level is chosen as the origin of the energy.

The density of states (DOS) of the present studied compounds, La_15_Ni_13_Bi_5_ and La_9_Ni_8_Sn_5_, are shown in Figure 5a,b, respectively. When compared to the electronic structure of previously reported LaNi_*y*_-based compounds,^56,57^ which are rather Ni-rich, in the significantly La-richer compounds La_15_Ni_13_Bi_5_ and La_9_Ni_8_Sn_5_ significant electron transfer from La to Ni occurs as Bader charge analysis reveals, *c.f*. Table 3, with a Bader charge of about +1.2 electron for La. The localized, empty 4*f*-La states are located around 2 eV above the Fermi level. Thanks to the given electrons from La, the 3*d*-Ni bands are almost entirely filled, and a pseudogap arises at the Fermi level. Therefore, the DFT calculation converges in a Pauli paramagnetic state, even if ferromagnetic initialization is forced as a starting set.

**Table 3:** Average Bader Electronic Charge by Atom for La_15_Ni_13_Bi_5_ and La_9_Ni_8_Sn_5_

	Atom		
Compound	La	Ni	Bi/Sn
La_15_Ni_13_Bi_5_	–1.18	+0.82	+1.41
La_9_Ni_8_Sn_5_	–1.16	+0.80	+0.80

**Figure 6:**
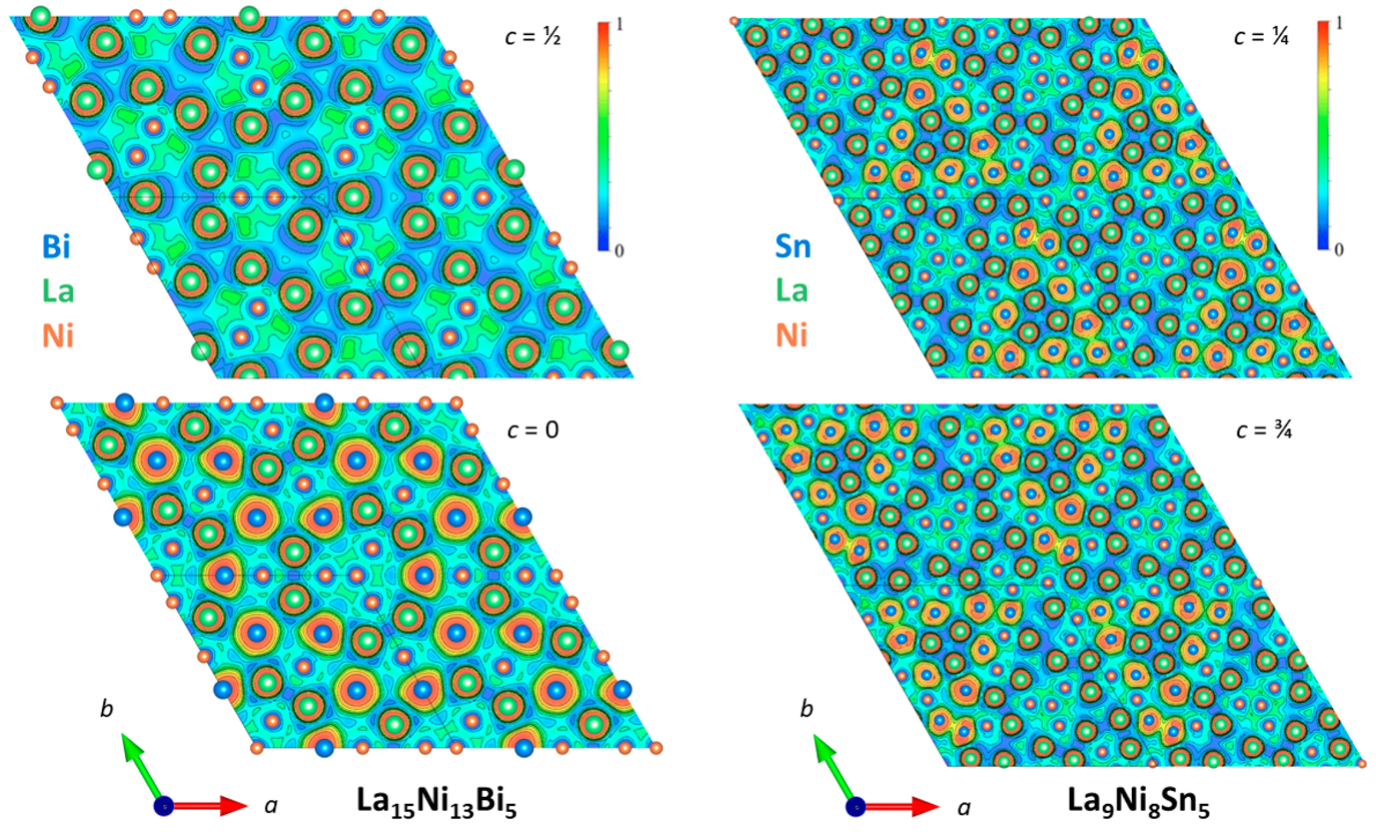
ELF representation of La_15_Ni_13_Bi_5_ and an ordered approximation of La_9_Ni_8_Sn_5_ in two different planes perpendicular to the *c* axis. Warm color (red) areas indicate a strongly localized pair-electrons zone (probability of presence close to 1), whereas cold color (blue) represents nonlocalized electrons.

The differences in the electronic structure of La_15_Ni_13_Bi_5_ and La_9_Ni_8_Sn_5_ are mainly due to different *p*-elements. Whereas the 6*s* band of Bi is found at −10 eV below Fermi level *E*_F_, the 6*p*-Bi states interact well with La and Ni states in the range from −4 eV to *E*_F_. Compared to that, the 5*s* and 5*p* states of Sn are separated by a lower gap due to smaller scalar relativistic effects in Sn compared to Bi with a broader bonding 5*s* band of Sn located at about −8 eV featuring some interaction with Ni states, comparable to those found in compounds such as LaNi_4.75_Sn_0.25_.^56^ The 5*p* states of Sn are located at higher energies, interacting with the conduction *d*-metal bands, similar to those of 6*p*-Bi. Since bismuth is more electronegative than tin, it attracts more electrons: Bi and Sn present a Bader charge of 1.4 and 0.8 electrons, respectively. The difference in electronic band dispersion, or here, energetic dispersion of the DOS, can not only be attributed to the different energetic levels of the states but, with respect to the *p*-states of the respective *p*-element, the ability to interact with its local environment, i.e., its next neighbors in the structure. In fact, in the case of La_15_Ni_13_Bi_5_, Bi is completely isolated inside of La-prims, whereas Sn also allows mixing with Ni in La_9_Ni_8_Sn_5_ and a broader range of interactions. This is supported by the ELF plot, as shown in Figure 6.

The ELF function exhibits maxima at the most probable positions of localized electron pairs, and each maximum is surrounded by a basin (isosurface not shown) in which there is an increased probability of finding electrons. In fact, when high values for ELF are chosen, one can identify the electron pair domains that form the bonds. There is no maximum area between the atoms, since there are no covalent bonds here. The system is characterized by metallic bonds with a rather low ELF and by the narrow width of the localization windows around atoms.^58^ The last valence electron shell of La is missing due to charge transfer. We can see that La contributes a smaller part of the bonding electrons than Ni and the *p*-element, as shown by the very localized pair-electron region located around the La and Bi/Sn atoms. The localized area of Sn is less important than that of Bi due to its interaction with Ni as discussed in the DOS analysis. Ni is surrounded by a nonlocalized electron region and forms a preferential metallic bond with other elements such as Sn, which supports the honeycomb framework. One can observe a localized region between some tin atoms, resulting in a sparse Sn–Sn dumbbell appearance with one covalent bond, but this behavior could not be generalized to all Sn–Sn bonds. In fact, it results from the random atom distribution considered in the chosen periodic supercell, constrained by the partial occupation of the Sn1 site.

**Figure 7:**
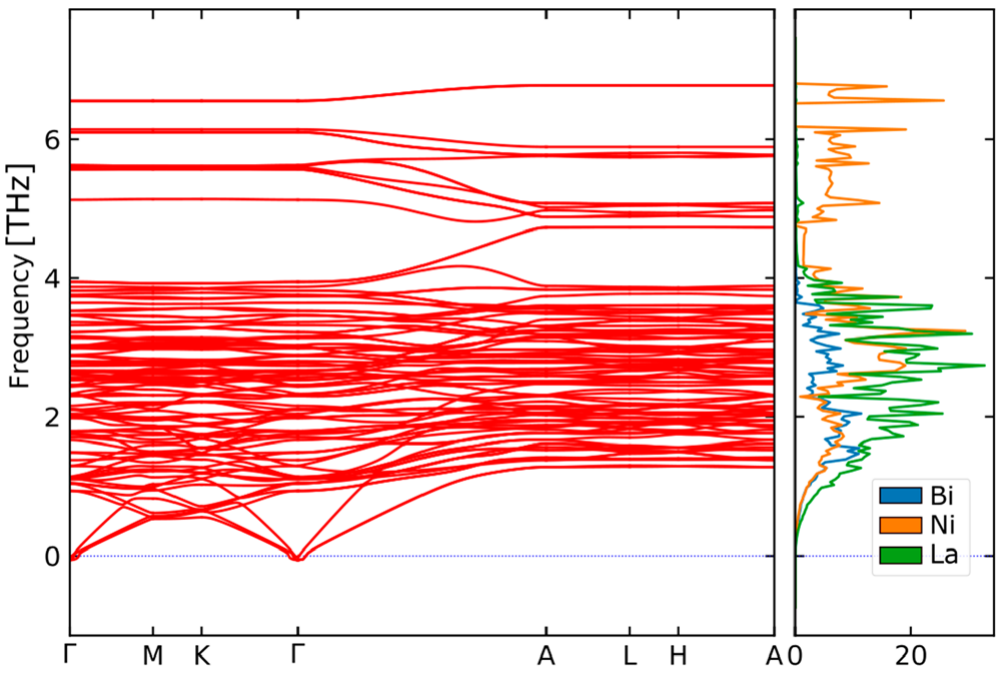
Phonon dispersion curves for La_15_Ni_13_Bi_5_.

Both compounds are stable relative to the pure elements, with a negative heat of formation of −54.00 and −47.23 kJ/mol for La_15_Ni_13_Bi_5_ and La_9_Ni_8_Sn_5_, respectively. Moreover, phonon calculations have been performed for the ordered La_15_Ni_13_Bi_5_ structure. The phonon bands are shown in Figure 7 with associated acoustic bands from heavier elements and optical flat bands associated with Ni elements at around 6 THz. The vibrational dispersion curves present no imaginary frequency, yielding the confirmation of the mechanical stability of the honeycomb network in the La_15_Ni_13_Bi_5_ phase.

### 3.3 Magnetic Behavior

**Figure 8:**
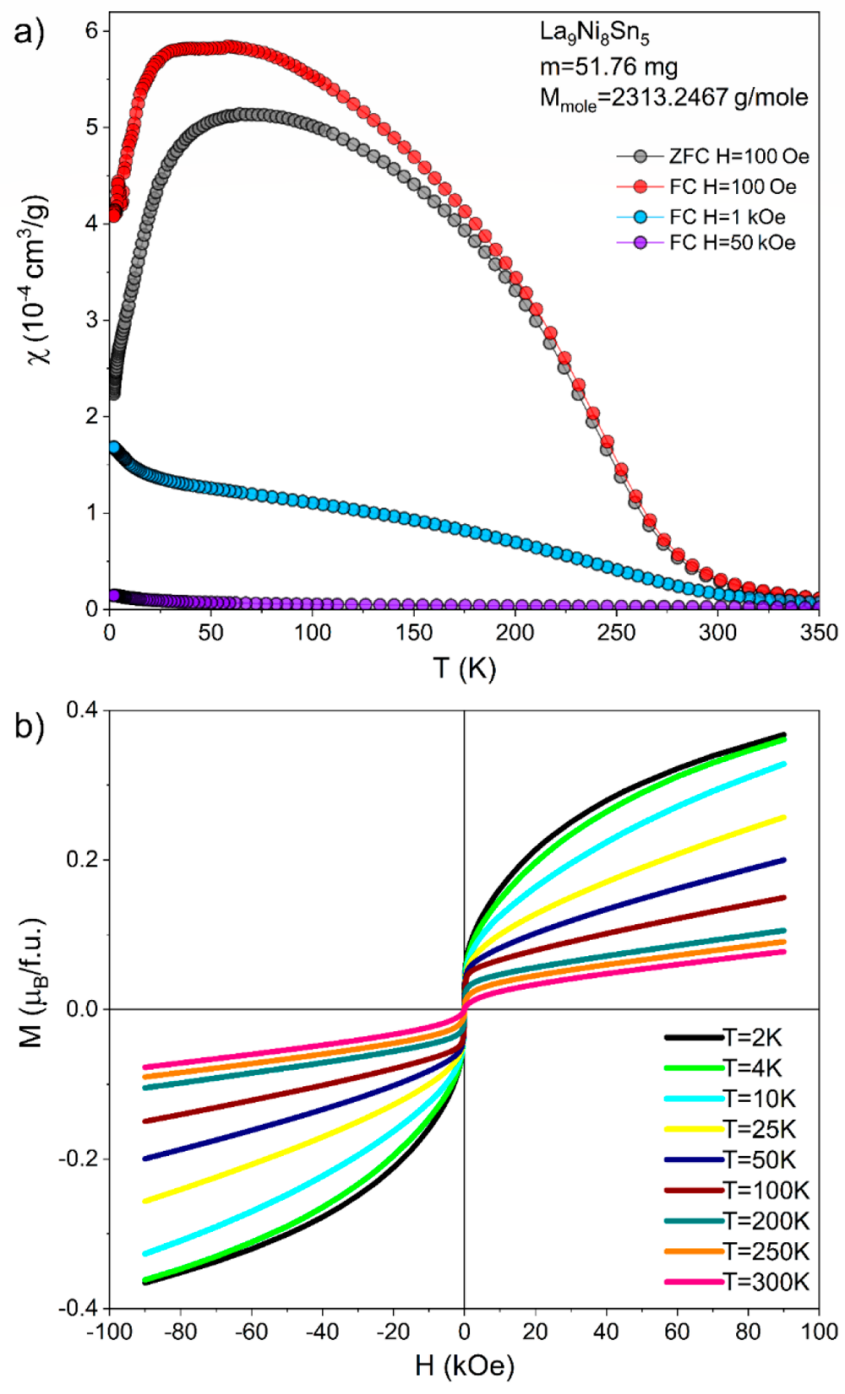
Magnetic properties of La_9_Ni_8_Sn_5_: susceptibility measured at different magnetic fields (a) and isothermal magnetization (b).

The results from the magnetic measurements for La_9_Ni_8_Sn_5_ are presented in Figure 8. The magnetic susceptibility at 100 Oe shows a complex behavior with a plateau between 50 and 100 K, while at lower temperatures both FC and ZFC curves go to lower values. For higher fields the above behavior is suppressed, being a mark of weak magnetism. Inverse magnetization does not follow the Curie–Weiss law (even when modified) for fields less than 1 kOe. Applying a high magnetic field partially linearizes the inverse magnetic susceptibility, as presented in Figure S7. Extraction of a ferromagnetic impurity by the Honda–Owen method also does not show results that can be easily interpreted (Figure S7). Therefore, it may be concluded that the magnetic properties of the compound are presumably weak or paramagnetic, overlaid on magnetism originating from spurious phases. According to our XRD/EDX studies, small amounts of impurities were detected (Figures S1 and S2). For example, the La_2_Ni_7_ is an itinerant weak antiferromagnet with an ordering temperature of 51 K (wAFM).^59^ LaNi_3_ was detected as the other impurity. According to the literature, it shows weak itinerant magnetism below 28 K, while neutron diffraction showed no magnetic contribution at all.^60^ Additionally, EDX results indicated extremely small precipitations of Ce (cerium is a common impurity encountered in La, even in commercial 99.9% La). As Ce shows a complex magnetic ordering at 12.5 K, the upturn of susceptibility could be associated with Ce precipitations. Small ferromagnetic components visible for all isothermal magnetization curves are presumably related to small amounts of Ni precipitations. However, it is worth mentioning that no exchange bias was noticed for the curves, which may originate from the nanostructural morphology of the Ni precipitations. It is also evident that the shape of FC/ZFC curves, with broad anomaly, resembles behavior often seen for low-dimensional magnets.^61^

**Figure 9:**
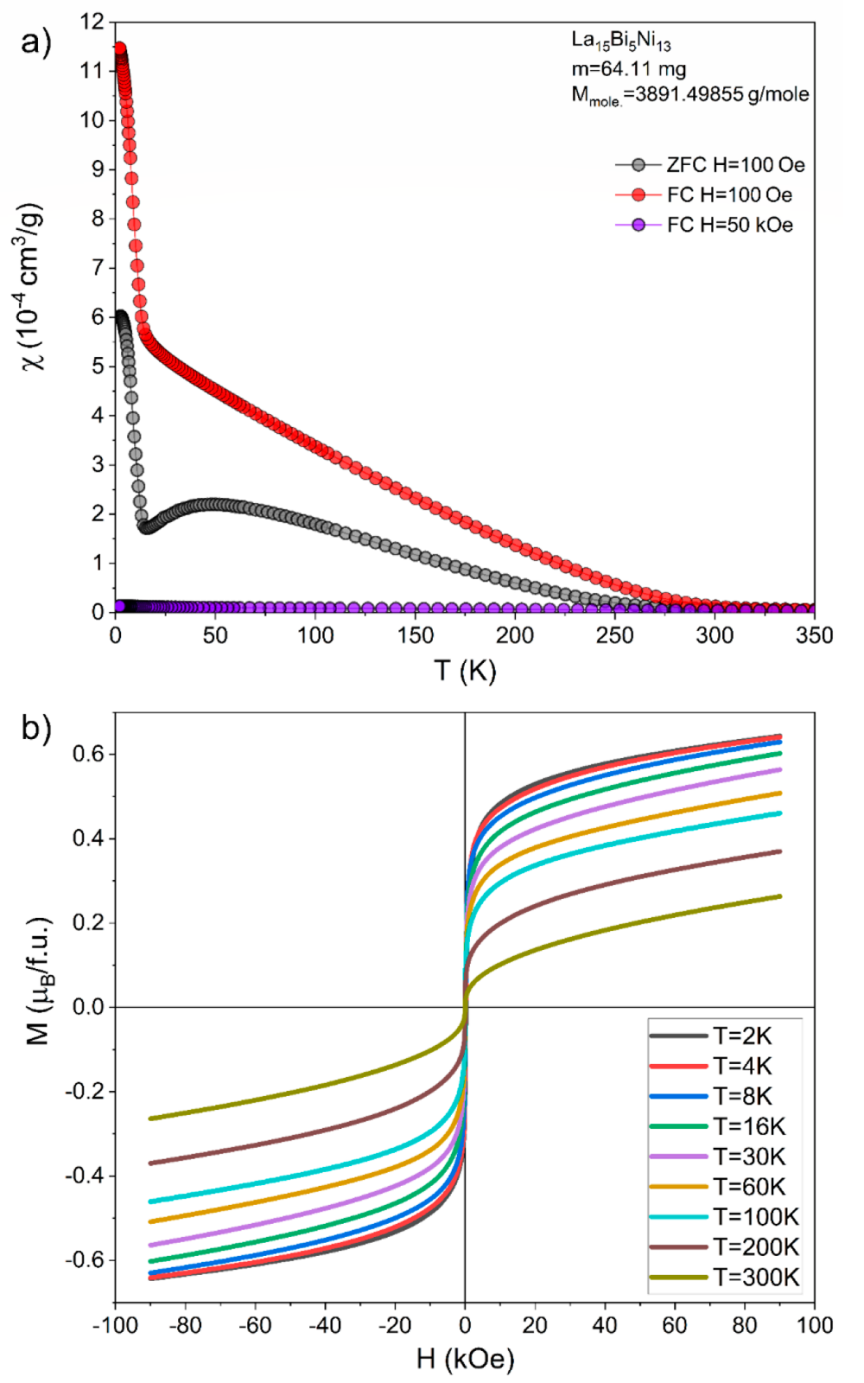
Magnetic properties of La_15_Ni_13_Bi_5_: susceptibility measured at different magnetic fields (a) and isothermal magnetization (b).

For the La_15_Ni_13_Bi_5_ compound, magnetic susceptibility vs temperature is shown in Figure 9. As is apparent, in the 25–300 K temperature range, the magnetic susceptibility is dominated by small ferromagnetic components. It can be seen in the inset to Figure 9, where isothermal magnetization curves show a ferromagnetic component between 2 and 300 K. The ferromagnetic component most likely originates from very small Ni-rich precipitates (due to alloy decomposition/oxidation on air during the preparation of the sample for magnetic measurement). Interestingly, at low temperatures, below 10 K ZFC and FC curves show some anomalous behavior. This presumably originates from La_1–*x*_Ce_*x*_Bi (CeBi and pure Ce impurities, which order magnetically at 26 and 12.5 K, respectively).^62,63^

Inverse magnetic susceptibility measured at high magnetic fields (50 kOe) did not show Curie–Weiss behavior; therefore, estimating the effective magnetic moment was not possible. Nonetheless, a broad maximum in the ZFC curve suggests some dimensionally restricted magnetism.

## 4 Conclusion

The new ternary intermetallics La_15_Ni_13_Bi_5_ and La_9_Ni_8_Sn_5_ have been synthesized, and their properties investigated. The crystal structure of La_15_Ni_13_Bi_5_ is characterized by pseudo-1D honeycomb-based motifs of Ni atoms surrounded by La atoms. La_9_Ni_8_Sn_5_ is characterized by a honeycomb-based polyanionic Ni/Sn network encapsulating Sn-centered polyhedra. The latter structure exhibits substantial disorder in the area around the *c* axis. Honeycomb-based motifs observed in both compounds extend along the *c* axis, in contrast to planar or pseudoplanar layers in graphite or similar inorganic structures where such constructs are normal to the 3(6)-fold axis. The core building fragments of the honeycomb motifs bear some analogies to organic aromatic molecules like triptycene, while the pseudo-1D construct observed in La_15_Ni_13_Bi_5_ is, to the best of our knowledge, unique in the solid state.

Magnetometric measurements revealed that La_9_Ni_8_Sn_5_ likely exhibits paramagnetism or weak magnetism with magnetic response dominated by spurious La–Ni phases. On the other hand, La_15_Ni_13_Bi_5_ is probably a Pauli paramagnet with magnetic properties biased by ferromagnetic impurities; however, some sign of low-dimensional magnetism could be noticed in the ZFC curve.

DFT and phonon calculations confirm the mechanical stability of these honeycomb constructs explained by a metallic bond with an electronic charge transfer from La to Ni and Bi/Sn atoms.

## Supporting Information Available

Additional crystallographic information; Figures S1–S7 (PDF)
