# Supplementary material for: Honeycomb Constructs in the La–Ni Intermetallics: Controlling Dimensionality via p-Element Substitution
Source: Inorg Chem. 2023 Sep 7;62(37):14843–51. doi: 10.1021/acs.inorgchem.3c00502 (PMC10521015; doi:10.1021/acs.inorgchem.3c00502)
Supplement: Supplementary file 1 — ic3c00502_si_001.pdf [file ic3c00502_si_001.pdf]

Supporting Information

Honeycomb constructs in the La-Ni intermetallics: Controlling dimensionality via *p*-element substitution

Vitalii Shtender[1], Volodymyr Smetana[2], Jean-Claude Crivello[3], Łukasz Gondek[4], Janusz Przewoźnik[4], Anja-Verena Mudring[2], Martin Sahlberg[1]

[1]*Department of Chemistry – Ångström Laboratory, Uppsala University, Box 538, 751 21, Uppsala, Sweden*

[2]*Department of Materials and Environmental Chemistry, Stockholm University, Svante Arrhenius väg 16c, 10691 Stockholm, Sweden*

[3]*Univ Paris Est Creteil, CNRS, ICMPE, UMR7182, 2 rue Henri Dunant, 94320 Thiais, France*

[4]*AGH University of Science and Technology, Faculty of Physics and Applied Computer Science, Mickiewicza 30, 30-059, Krakow, Poland*

*Corresponding author: vitalii.shtender@kemi.uu.se

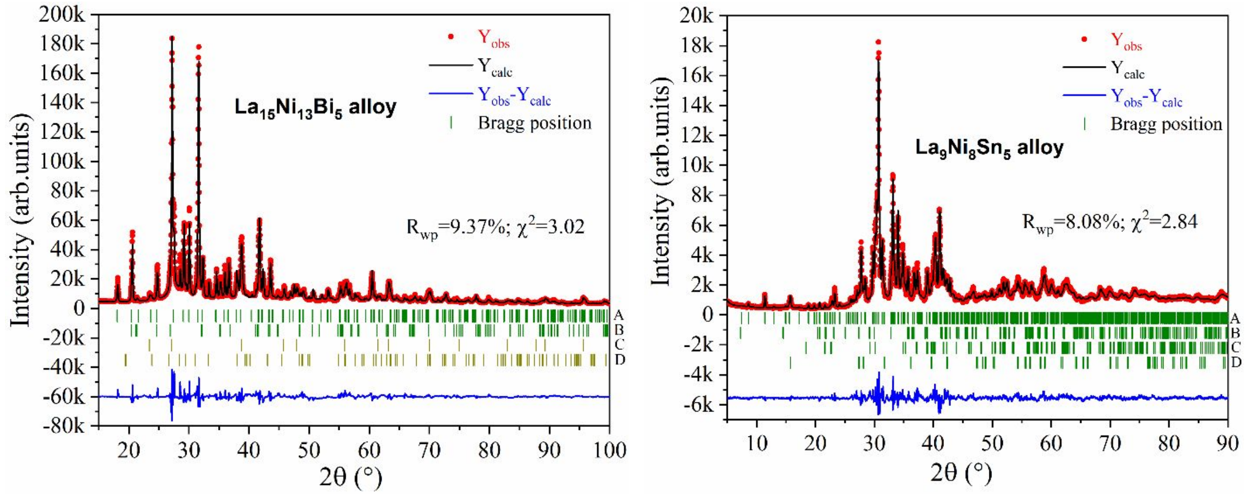

A-74.5% $La_{15}Ni_{13}Bi_5$, B-7.2% $LaNi_3$, C-15.1% LaBi, D-3.2% La_2Bi

A-83.2% $La_9Ni_8Sn_5$, B-10.2% La_2Ni_7, C-4.4% La_2Ni_3, D-2.2% $La(OH)_3$

Figure S1. The XRD patterns with the results of the Rietveld refinement for Bi- and Sn-based alloys. Bragg positions for refined phases are outlined with capital letters and the resulting phase content of different phases are presented below the corresponding picture.

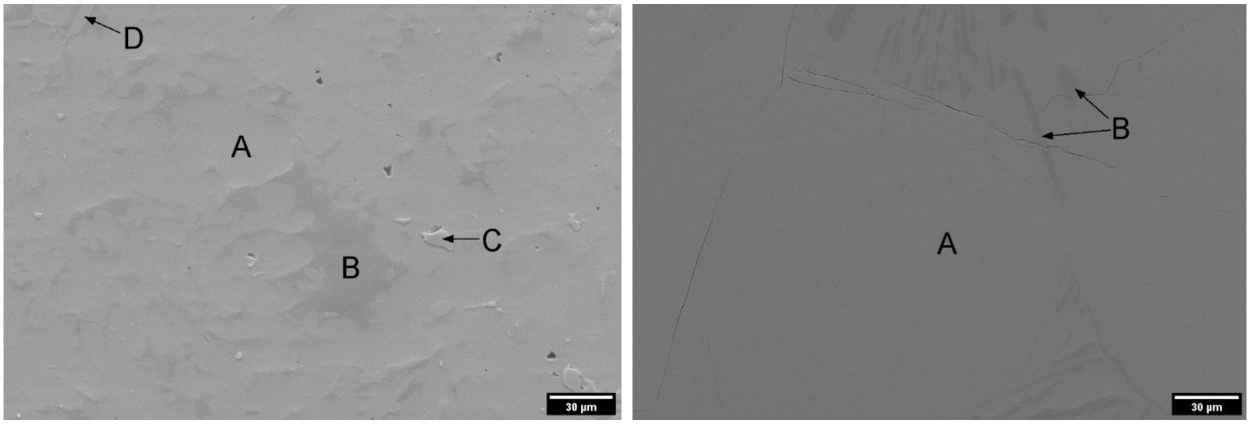

A-$La_{15}Ni_{13}Bi_5$, B-$LaNi_3$, C-LaBi, D-La_2Bi A-$La_9Ni_8Sn_5$, B-La_2Ni_7

Figure S2. SEM images [backscattered electron (BSE) model] for Bi- and Sn-based alloys. Different regions outlined with capital letters were scanned with EDX detector and the resulting compositions of different detected phases are presented below the corresponding picture.

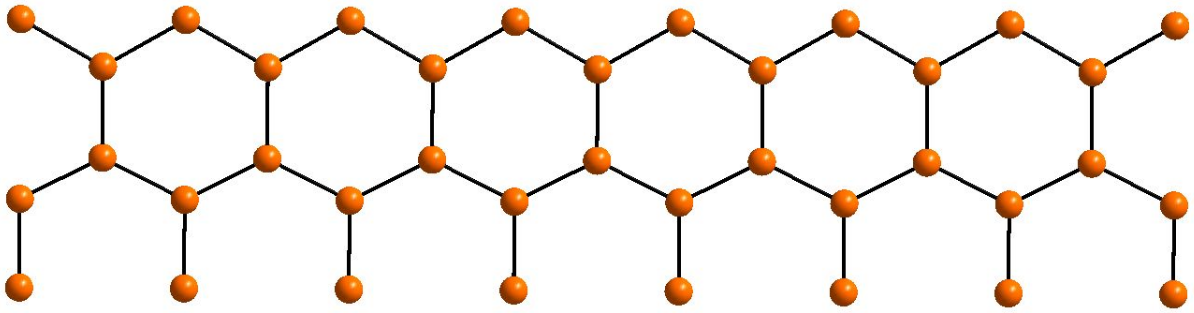

Figure S3. Single blade of the Ni stick. The lower (central) hexagons are missing a vertex.

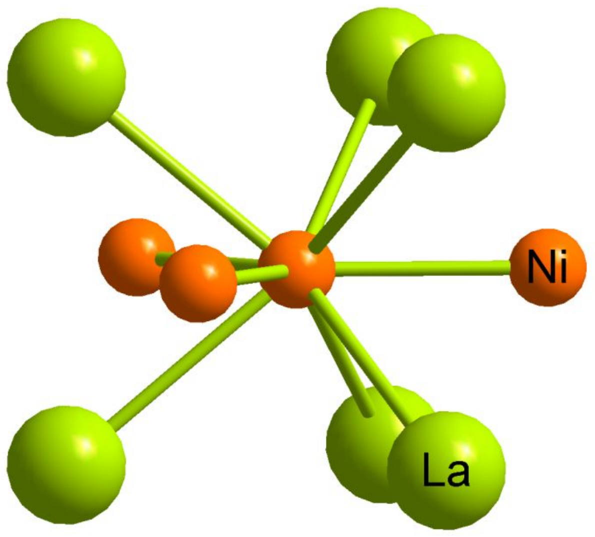

Figure S4. Ni@Ni_3La_6 tricapped hexagonal prisms in the crystal structure of $La_{15}Ni_{13}Bi_5$.

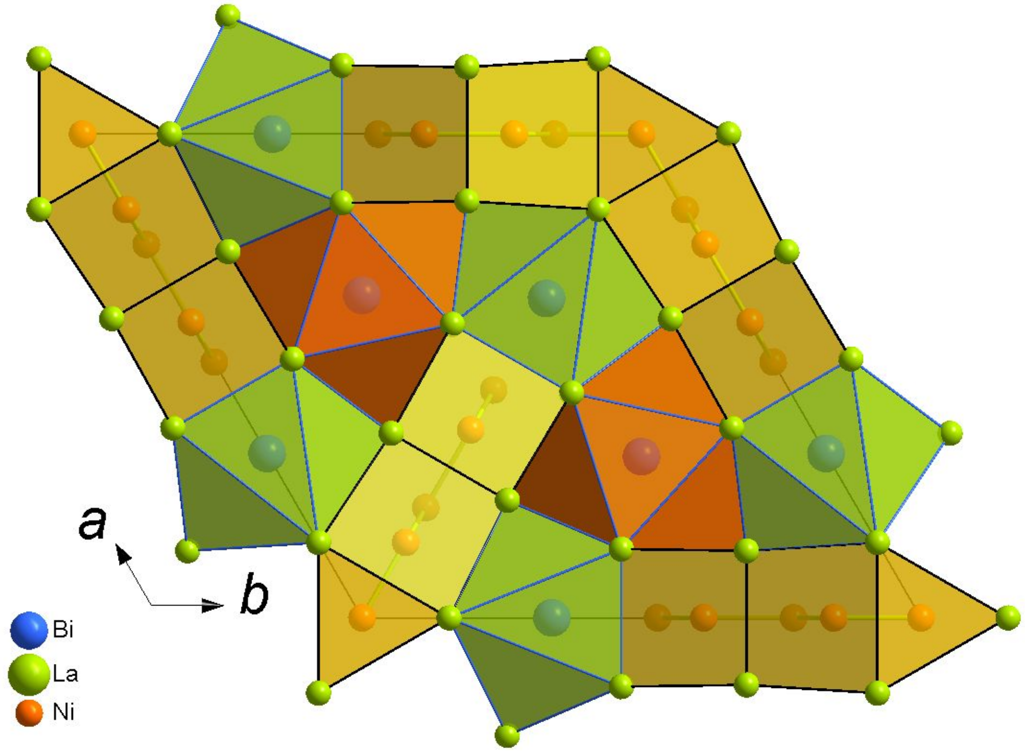

Figure S5. Polyhedral representation of the crystal structure of $La_{15}Ni_{13}Bi_5$. Bi@La_9 tricapped trigonal prisms are orange, Bi@La_8 bicapped trigonal prisms – green, and Ni@La_6 trigonal prisms – dark yellow.

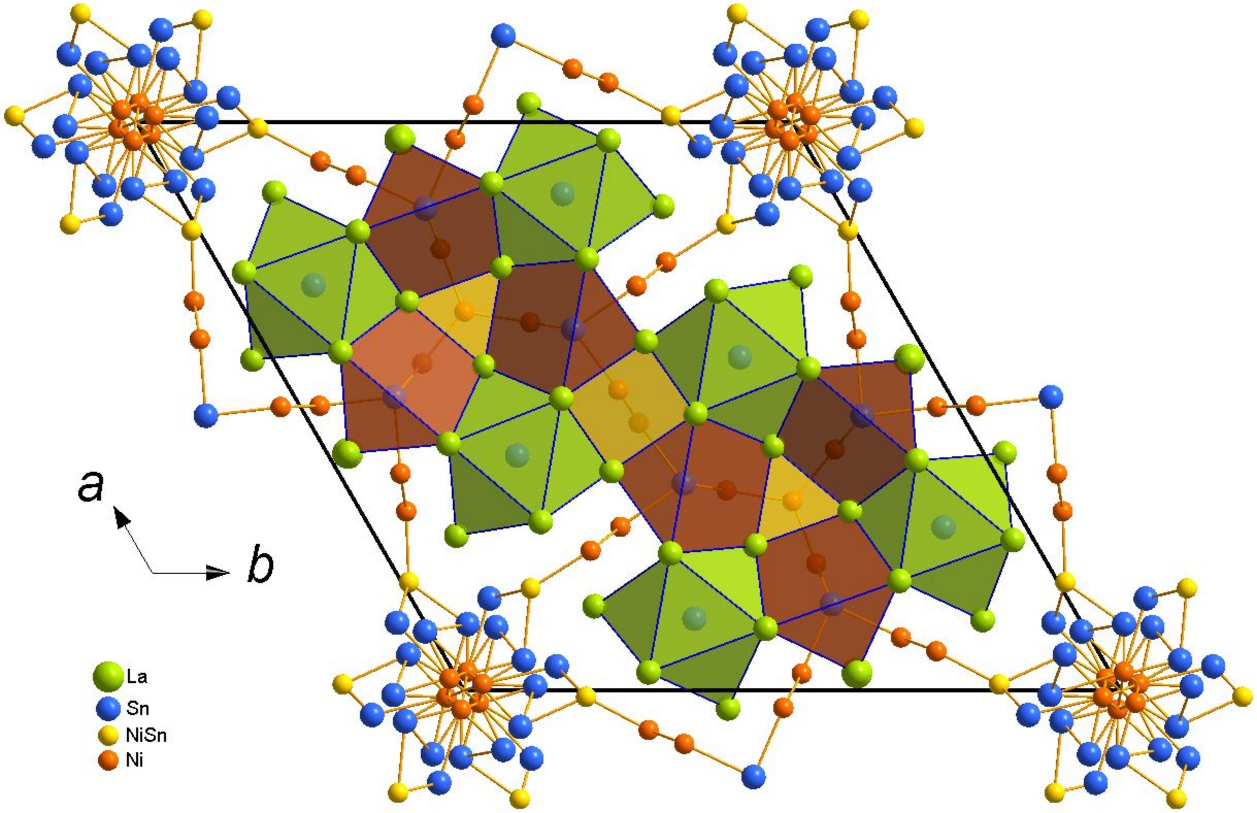

Figure S6. Polyhedral representation of the ordered part of the crystal structure of $La_9Ni_8Sn_5$. Sn@La_9 tricapped trigonal prisms are green, Sn@La_8 bicapped trigonal prisms – brown, and Ni@La_6 trigonal prisms – dark yellow.

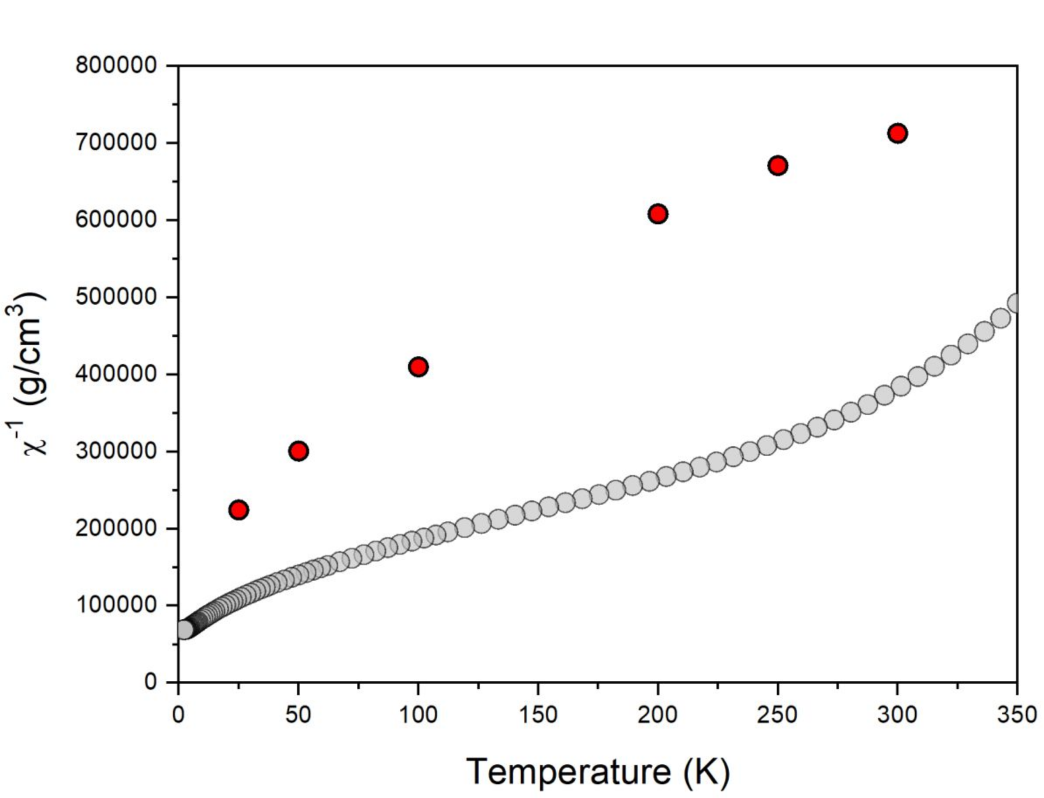

Figure S7. Inverse magnetic susceptibility (H=5T) of $La_9Ni_8Sn_5$ (gray points). Red points mark inverse susceptibility after subtracting the ferromagnetic impurities by Honda-Owen method.
